# First report of carbapenemase-producing bacteria from poultry farms in the United Arab Emirates: an emerging One Health threat

**DOI:** 10.3389/fvets.2026.1847960

**Published:** 2026-09-24

**Authors:** Hazim O. Khalifa, Temesgen Mohammed, Mushal Allam, Afra Abdalla, Mohamed-Yousif Ibrahim Mohamed, Glindya Bhagya Lakshmi, Febin Anes, Farah Al-Marzooq, Ihab Habib

**Affiliations:** 1 Department of Veterinary Medicine, College of Agriculture and Veterinary Medicine, United Arab Emirates University, Al Ain, United Arab Emirates; 2 Department of Genetics and Genomics, College of Medicine and Health Sciences, United Arab Emirates University, Al Ain, United Arab Emirates; 3 Veterinary Control Section, Animal Welfare Department, Municipal Services Sector, Abu Dhabi, United Arab Emirates; 4 ASPIRE Research Institute for Food Security in the Drylands (ARIFSID), United Arab Emirates University, Al Ain, United Arab Emirates; 5 Department of Medical Microbiology and Immunology, College of Medicine and Health Sciences, United Arab Emirates University, Al Ain, United Arab Emirates

**Keywords:** animal-to-human transmission, antimicrobial resistance, *bla*_NDM-5_, *bla*_POM-1_, carbapenemases, chicken cecum, *K. pneumoniae*, *Metapseudomonas otitidis*

## Abstract

**Background:**

Carbapenemase-producing bacteria represent a critical global public health threat, yet their presence and molecular characteristics in food animal production systems remain poorly understood. This study provides the first comprehensive report of carbapenemase-producing bacteria isolated from poultry farms in the United Arab Emirates, offering important One Health insights into emerging antimicrobial resistance risks at the human–animal–environment interface.

**Methods:**

A total of 77 samples were collected from broiler farmhouses and slaughterhouses belonging to two major poultry companies in Al Ain, United Arab Emirates, between June 2023 and April 2024. Isolates were screened on MacConkey agar supplemented with meropenem, and carbapenemase production was confirmed using the modified carbapenem inactivation method. Antimicrobial susceptibility testing was performed, followed by whole-genome sequencing for species identification, resistome and virulome characterization, plasmid profiling, and comparative genomic analysis.

**Results:**

Three isolates (3.9%) were confirmed as carbapenemase producers, two of which exhibited multidrug resistance. Whole-genome sequencing identified one *Klebsiella pneumoniae* ST11 KL105 isolate carrying *bla*_NDM-5_ and two *Metapseudomonas otitidis* isolates harboring the intrinsic carbapenemase *bla*_POM-1_. The *K. pneumoniae* isolate contained multiple plasmid replicons and a broad resistome. Virulence analysis revealed a complete yersiniabactin locus, enterobactin operon, *ompA*, and ECP-like pilus genes. Comparative genomic analysis demonstrated that the *bla*_NDM-5_ genetic environment was highly conserved and nearly identical to that of a human ST11 isolate recovered in India in 2019. The two *M. otitidis* isolates showed a highly syntenic chromosomal *bla*_POM-1_ locus and clustered closely with a human isolate from the United States (2016).

**Conclusion:**

This study documents the presence of carbapenemase-producing bacteria in poultry production systems in the UAE, including a high-risk *bla*_NDM-5_–harboring *K. pneumoniae* ST11 clone. The close genomic relatedness to human clinical isolates highlights the potential for cross-sectoral dissemination and underscores the importance of integrated genomic surveillance across animal, human, and environmental reservoirs.

## 1 Introduction

Antimicrobials play a crucial role in tackling infectious diseases and are essential for high-risk medical procedures like organ transplants, cancer treatments, and major surgeries. Unfortunately, the rampant overuse and misuse of these vital drugs have led to the rise of antimicrobial resistance (AMR), which is now recognized as one of the most pressing global health challenges. AMR occurs when microorganisms develop the ability to resist the effects of antimicrobial agents, thereby reducing treatment effectiveness over time (1). This ongoing crisis threatens progress in both human and veterinary medicine, food production, and public health. Unlike sudden infectious outbreaks, AMR has been steadily increasing since the early days of antimicrobial use and is now recognized by the World Health Organization as a major global health threat (1). The rise of drug-resistant pathogens threatens these critical advancements, affecting animal health and agricultural productivity and creating widespread consequences for food systems. Some estimates suggest that if a strong action not taken, AMR could lead to as many as 10 million deaths each year by 2050, along with significant socioeconomic repercussions from longer illnesses, higher treatment costs, and decreased workforce productivity (2).

Carbapenems, such as meropenem, imipenem, and ertapenem, are among the most potent β-lactam antibiotics and are often considered last-resort options for treating severe or multidrug-resistant bacterial infections in humans (3, 4). It is important to note that carbapenems are not approved for routine use in food-producing animals or agriculture, which makes the detection of carbapenem-resistant organisms in these environments particularly concerning (5, 6). Resistance to carbapenems in Gram-negative bacteria can develop through various mechanisms, such as changes in membrane permeability, increased activity of efflux pumps, or modifications in penicillin-binding proteins (5–7). However, the most clinically significant mechanism is the production of carbapenemases—enzymes that can break down carbapenems and other β-lactams (7). These enzymes belong to major families like KPC, NDM, VIM, IMP, and OXA-48-like, and they are often found on mobile genetic elements (7). This means they can be transferred quickly between different bacterial species, as well as among animals, the environment, and humans, which heightens the One Health risk associated with carbapenem resistance.

It is particularly alarming to find carbapenemase-producing Enterobacterales (CPE) and other Gram-negative pathogens in livestock and food chains (3, 5, 6). These organisms have been reported in various countries and can easily spread between animals, food products, the environment, and human populations (5, 6). Zoonotic bacteria such as *Escherichia coli, Klebsiella pneumoniae*, and *Pseudomonas* spp. can serve as reservoirs for carbapenemase genes, facilitating their spread through direct contact, contaminated meat, or cross-contamination during food handling (3, 5, 6). These bacteria often carry additional resistance traits, which add to the challenge of treating infections and controlling their spread.

Despite growing global concerns, there is a lack of data on carbapenemase-producing Gram-negative bacteria in the United Arab Emirates, especially in poultry farms. It is crucial to understand their presence, resistance traits, and potential transmission routes to guide antimicrobial stewardship and safeguard public health. This study aims to address a critical knowledge gap by conducting exploratory genomic surveillance of carbapenemase-producing Gram-negative bacteria in poultry production systems in the United Arab Emirates. Specifically, we sought to detect and characterize these organisms through phenotypic and whole-genome sequencing approaches, providing initial insights into their occurrence, resistome, and virulome profiles. In addition, comparative genomic analyses were performed to contextualize the identified isolates within globally circulating lineages, thereby highlighting potential cross-sectoral connections within a One Health framework, while not inferring direct transmission pathways.

## 2 Materials and methods

### 2.1 Sample details

A total of 77 poultry-associated samples were obtained over an eleven-month sampling campaign conducted between June 2023 and April 2024 in Al Ain City, United Arab Emirates. Samples were collected from broiler farmhouses and slaughterhouses operated by two large commercial poultry companies to capture variability across production systems and seasonal cycles. The sampling set consisted of 59 cecal droppings and 18 cecal content specimens. To ensure spatial and operational diversity, samples from the first company were collected from 18 poultry houses distributed across six farms, whereas samples from the second company originated from nine poultry houses across three farms. All sample collection, handling, and microbiological procedures were performed under strict aseptic conditions following established laboratory protocols to minimize the risk of contamination. Sterile, single-use consumables were employed throughout, and sample processing was conducted in a controlled laboratory environment with appropriate separation of pre- and post-culture steps. Sampling procedures and initial bacterial isolation followed protocols previously reported by our group (8), with the key modification that selective isolation of carbapenem-resistant organisms was performed using MacConkey agar supplemented with 1 μg/mL meropenem.

### 2.2 Phenotypic detection of carbapenemase production

Following selective culture, plates were incubated aerobically at 37 °C for 24 h. Colonies displaying distinct morphological characteristics were subcultured onto nutrient agar and incubated under the same conditions to obtain pure isolates. All recovered isolates were subsequently screened for carbapenemase activity using the modified carbapenem inactivation method (mCIM), as previously described (7). Briefly, a loopful of overnight bacterial culture was suspended in 2 mL of tryptic soy broth, vortexed, and incubated with a 10-μg meropenem disk at 37 °C for 4 h. The disk was then transferred onto Mueller–Hinton agar plates seeded with a standardized suspension (0.5 McFarland) of *Escherichia coli* ATCC 25922. After incubation for 18–24 h at 37 °C, carbapenemase activity was interpreted based on the size of the inhibition zone surrounding the meropenem disk. All phenotypic assays were performed under standardized laboratory conditions with appropriate aseptic procedures. Although selective culture and mCIM were performed following established protocols, certified carbapenemase-positive and -negative reference strains were not systematically included as external quality controls, which is acknowledged as a limitation of the study.

### 2.3 Antimicrobial sensitivity testing

Antimicrobial susceptibility profiles of mCIM-positive isolates were determined using the Kirby–Bauer disk diffusion method in accordance with Clinical and Laboratory Standards Institute (CLSI) guidelines (M100-S30, 2020) (9). *E. coli* ATCC 25922 was used for quality control. Antibiotic disks (HiMedia, Maharashtra, India) representing multiple antimicrobial classes were tested, including β-lactams (ampicillin, amoxicillin–clavulanic acid, cefoperazone, ceftriaxone, cefoxitin, meropenem, imipenem), quinolones (ciprofloxacin, nalidixic acid), aminoglycosides (gentamicin, amikacin), tetracycline, and chloramphenicol. Zone diameters were measured and interpreted using CLSI breakpoints.

### 2.4 Whole genome sequence (WGS) analysis

Whole-genome sequencing was performed on the three isolates confirmed as carbapenemase producers to validate phenotypic findings and enable in-depth genomic characterization. Genomic DNA was extracted using the Wizard® Genomic DNA Purification Kit (Promega, USA) following the manufacturer’s protocol. DNA quantity and purity were assessed using both ScanDrop2 and NanoDrop 1,000 spectrophotometers to ensure suitability for sequencing. Sequencing libraries were prepared using the Nextera XT kit (Illumina, USA), and paired-end sequencing (150 bp) was conducted on the Illumina NovaSeq platform by Novogene (UK). Raw reads underwent quality control, *de novo* assembly, and multilocus sequence typing using the Jekesa pipeline^1^. Assembled genomes were screened against the NCBI AMR Reference Gene Database for resistance determinants, with confirmation using ABRicate^2^. Plasmid replicons and virulence-associated genes were identified using tools available on the Galaxy Australia platform^3^ (10). Comparative BLAST analysis to identify the genetic environment of *bla*_NDM-5_ was performed using Easyfig (11).

For phylogenetic reconstruction, 42 draft genomes of *Metapseudomonas otitidis* and 538 draft genomes of *K. pneumoniae* ST11 harboring the *bla*_NDM-5_ gene (downloaded on 01 October 2025) were included. Core genome single-nucleotide polymorphisms (SNPs) were identified using Snippy v4.6.0^4^, followed by removal of recombinant regions with Gubbins v2.4.1 (12). Maximum-likelihood phylogenetic trees were constructed using IQ-TREE v2.4.0 (13), with automatic model selection, and tree visualization, annotation, and refinement were carried out using Evolview v3^5^.

## 3 Results

### 3.1 Bacterial isolation and carbapenemase confirmation

Among the 77 samples screened, three isolates (3.9%) were confirmed as carbapenemase-producing using the mCIM method. The three isolates were recovered from a cecal dropping sampled directly within a farmhouse. Antimicrobial susceptibility testing by disk diffusion showed that these isolates were resistant to multiple antibiotics, including carbapenems (Table 1). Two isolates (268MR1 and 377MR1) met the criteria for multidrug resistance, exhibiting resistance to at least three antimicrobial classes. Whole-genome sequencing analysis identified one *K. pneumoniae* isolate (377MR1) harboring the *bla*_NDM-5_ gene and two *Metapseudomonas otitidis* isolates (201MP1 and 268MR1) carrying the *bla*_POM-1_ gene (Table 1).

**Table 1:** Phenotypic and genotypic characterization of carbapenemase producing Gram-negative isolates from poultry.

Strain	Bacterial identification	MLST	Resistance phenotype		Detected resistant genes			Detected plasmids
			Carbapenems	Other antibiotics	Carbapenemases	Other β-lactams	Other resistant genes	
377MR1	*Klebsiella pneumoniae*	ST11	MEM, IPM	AMP, AMC, CFP, CRO, FOX, CIP, NAL, TET, GEN, AMK	*bla*_NDM-5_	*bla*_SHV-182_, *bla*_TEM-1B_, *bla*_OXA-1_	*aac(6′)-Ib10*, a*ph(3′)-Ia*, *fosA6*, *mph(A)*, *qnrB1*, *catB3*, *sul1*, *tet(A)*, *dfrA12*, *dfrA14*, *rmtB*, *aadA2*, *oqxA*, *oqxB*	IncFIB(K), IncFII(K), IncR, Col(MG828)
201MP1	*Metapseudomonas otitidis*		MEM, IPM	AMP, AMC, CFP, CRO, FOX, CIP, NAL	*bla*_POM-1_	*bla*_LCR-1_	*qnrVC1*, *sul1*, *aadA10*, *tmexD4*	ND
268MR1	*Metapseudomonas otitidis*		MEM, IPM	AMP, AMC, CFP, CRO, FOX, CIP, NAL, CHL	*bla*_POM-1_	*bla*_LCR-1_	*qnrVC1*, *sul1*, *aadA10*, *tmexD4*	ND

AMP, ampicillin; CRO, ceftriaxone; FOX, cefoxitin; CFP, cefoperazone; CHL, chloramphenicol; NAL, nalidixic acid; GEN, gentamicin; AMK, amikacin; CIP, ciprofloxacin; ND, Not detected.

### 3.2 Whole genome sequencing–based detection of antimicrobial resistance genes

Full genetic characterization of the carbapenemase-producing isolates revealed notable diversity in species, sequence types, and associated resistomes. The *K. pneumoniae* isolate 377MR1 (ST11) carried the class B metallo-β-lactamase *bla*_NDM-5_, explaining its resistance to meropenem and imipenem. In addition, this strain harbored multiple β-lactamase genes (*bla*_SHV-182_, *bla*_TEM-1B_, and *bla*_OXA-1_) and a broad array of acquired resistance determinants, including aminoglycoside-modifying enzymes (*aac(6′)-Ib10*, *aph(3′)-Ia*, *rmtB*, *aadA2*), quinolone resistance genes (*qnrB1*, *OqxA*, *OqxB*), macrolide resistance *(mph(A))*, fosfomycin resistance (*fosA6*), phenicol resistance (*catB3*), sulfonamide resistance (*sul1*), tetracycline resistance *(tet(A))*, and trimethoprim resistance genes (*dfrA12*, *dfrA14*) (Table 1).

Two *M. otitidis* isolates (201MP1 and 268MR1) also exhibited carbapenem resistance mediated by the intrinsic chromosomal *bla*_POM-1_ carbapenemase, accompanied by the β-lactamase *bla*_LCR-1_. Both strains additionally carried the efflux pump gene *tmexD4*, which is associated with decreased susceptibility to tigecycline and contributes to multidrug efflux. They also harbored other resistance determinants, including *qnrVC1* (quinolone resistance), *sul1* (sulfonamide resistance), and *aadA10* (aminoglycoside resistance).

### 3.3 Plasmids and virulence genes detection

Plasmid analysis revealed the presence of multiple incompatibility groups in *K. pneumoniae* isolate 377MR1, which carried IncFIB(K), IncFII(K), IncR, and Col(MG828) plasmid replicons. In contrast, no plasmid replicons were detected in isolates *M. otitidis* 201MP1 and 268MR1, suggesting that their resistance determinants are likely chromosomally encoded rather than plasmid borne.

Analysis of virulence determinants revealed that the *K. pneumoniae* isolate 377MR1 harbored a diverse repertoire of virulence genes (Table 2). The strain carried a complete yersiniabactin siderophore cluster (*ybt* and *irp* genes), strongly associated with enhanced iron acquisition and increased pathogenic potential. In addition, genes from the enterobactin siderophore system (*fepC, entA, entB*) were detected, alongside ompA, a key outer membrane protein involved in adhesion and serum resistance. The isolate also possessed multiple genes from the *E. coli* common pilus (ECP)-like adhesin system, supporting its potential for epithelial attachment and biofilm formation.

**Table 2:** Virulence factors present in *K. pneumoniae* isolate 377MR1.

Virulence system	Genes detected	Function/importance
Yersiniabactin siderophore cluster (ICEKp-like)	*ybtS, ybtX, ybtQ, ybtP, ybtA, irp2, irp1, ybtU, ybtT, ybtE, fyuA*	High affinity siderophore system for iron acquisition; strongly associated with increased virulence and invasive disease (e.g., bacteremia, deep-seated infections).
Enterobactin siderophore system	*fepC, entB, entA*	Core siderophore system for iron uptake; contributes to growth and survival in iron-limited host environments.
Outer membrane protein	*ompA*	Outer membrane protein A; involved in adhesion, serum resistance, and interaction with host cells → important virulence factor in *Klebsiella*.
*E. coli* common pilus (ECP)–like adhesin system	*ykgK/ecpR, yagZ/ecpA, yagY/ecpB, yagX/ecpC, yagW/ecpD, yagV/ecpE*	Fimbrial adhesin system mediating attachment to host epithelial surfaces; contributes to colonization and biofilm formation.

Virulence profiling of the *M. otitidis* isolates 201MP1 and 268MR1 revealed highly similar virulence gene repertoires (Table 3). Both isolates carried a complete Type VI secretion system (HSI-I), enabling interbacterial competition and secretion of cytotoxic effectors. In addition, each strain harbored a full complement of pyoverdine siderophore genes, essential for iron acquisition and oxidative stress survival, along with an extensive flagellar motility gene cluster supporting motility and host colonization. Genes involved in alginate biosynthesis, contributing to biofilm formation and immune evasion, were also present in both isolates. Furthermore, both strains carried multiple genes related to type IV pili, facilitating adhesion and twitching motility, as well as components of the Type II secretion system and LPS core biosynthesis, which together enhance secretion of virulence factors and maintain outer membrane integrity.

**Table 3:** Virulence genes identified in *Metapseudomonas otitidis* Isolate 201MP1 and 268MR1.

Virulence system	201MP1	268MR1	Function/importance
Type VI secretion system (T6SS, HSI-I cluster)	*icmF1/tssM1, dotU1, hsiJ1, hsiB1/vipA, hsiC1/vipB, hcp1, hsiF1, hsiG1, clpV1, vgrG1b*	*icmF1/tssM1, dotU1, hsiJ1, hsiB1/vipA, hsiC1/vipB, hcp1, hsiF1, hsiG1, clpV1, vgrG1b*	Injection of antibacterial and antieukaryotic effectors; interbacterial competition; virulence and environmental persistence
Pyoverdine siderophore system	*pvdL, mbtH-like, pvdS, pvdM, pvdN, pvdO, pvdH, pvdA*	*pvdL, mbtH-like, pvdS, pvdA, pvdH, pvdO, pvdN, pvdM*	High-affinity iron acquisition; essential for virulence, oxidative stress survival, and host colonization
Flagellar motility system	*flgC, flgF, flgG, flgH, flgI, fleQ, fleN, fliE, fliF, fliG, fliI, fliM, fliN, fliP, fliQ, fliR, flhA, motA*	*flgC, flgF, flgG, flgH, flgI, fleQ, fleN, fliE, fliF, fliG, fliI, fliM, fliN, fliP, fliQ, fliR, flhA, motA*	Swimming motility, host surface colonization, tissue invasion, activation of immune responses
Alginate biosynthesis (biofilm formation)	*algA, algF, algI, alg8, algD, algB, algR, algU, algC, algW*	*algA, algF, algI, alg8, algD, algU, algB, algR, algC, algW*	Production and regulation of alginate; formation of biofilms; protection against host immunity and antimicrobials
Type IV Pili (twitching motility/adhesion)	*pilT, pilU, pilG, pilH, pilI, pilJ, pilR, xcpA/pilD*	*pilT, pilU, pilG, pilH, pilI, pilJ, pilR, xcpA/pilD*	Twitching motility, adhesion to surfaces, initial biofilm formation
Type II secretion system (Xcp system)	*xcpQ, xcpR, xcpS, xcpT,*	*xcpQ, xcpR, xcpS, xcpT*	Secretion of extracellular enzymes and toxins, nutrient acquisition, host tissue damage
LPS core biosynthesis	*waaA, waaG, waaC, waaF*	*waaA, waaC, waaG, waaF*	Construction of lipopolysaccharide core; outer membrane stability; immune evasion

### 3.4 Genetic environment of *bla*_NDM-5_ and *bla*_POM-1_

Comparative genomic analysis of the *bla*_NDM-5_ genetic environment in *K. pneumoniae* isolate 377MR1 revealed a highly conserved multidrug-resistance module closely resembling that of the K22_GCF_040716055 isolate recovered from a human in 2019 from India (Figure 1). In 377MR1, *bla*_NDM-5_ was embedded within a characteristic gene cassette composed of IS*30*–*bla*_NDM-5_–*ble*_MBL_–*PRAI*–*DsbD*, flanked upstream by an IS*6* element and downstream by an IS*91*-like transposase. Alignment with the Indian isolate demonstrated nearly complete synteny and >98% nucleotide identity across the shared region (green shading), indicating that both strains harbor the same mobile genetic backbone responsible for the mobilization and stability of *bla*_NDM-5_.

**Figure 1:**
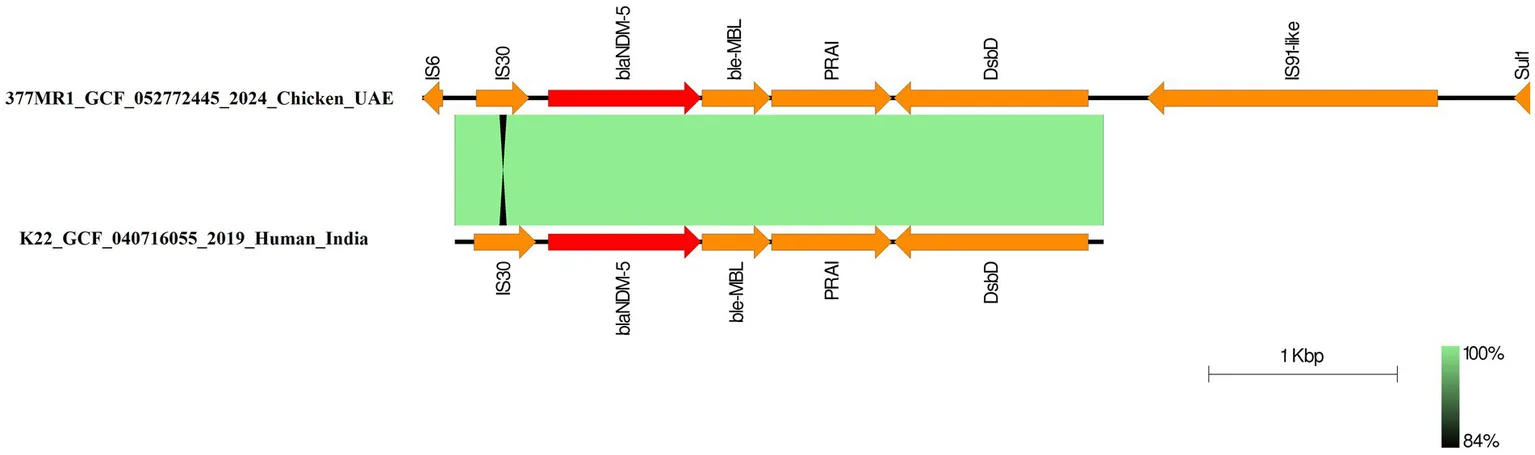
Genetic environment of *bla*_NDM-5_ in *Klebsiella pneumoniae* isolate 377MR1 compared with the human isolate K22_GCF_040716055 (India, 2019). The *bla*_NDM-5_ gene is embedded within a conserved IS*30*–*bla*_NDM-5_–*ble*_MBL_–PRAI–*dsbD* cassette, flanked by IS*6* and an IS*91*-like element. The region shows near-complete synteny and >98% nucleotide identity (green shading), indicating a shared mobile genetic backbone between animal and human isolates.

Comparative analysis of the chromosomal regions surrounding *bla*_POM-1_ in *M. otitidis* isolates 201MP1 and 268MR1 revealed an almost entirely conserved genomic organization (Figure 2). Both isolates exhibited a highly syntenic gene arrangement, with *bla*_POM-1_ positioned downstream of the conserved *mmhH*–*phnE* region and flanked by housekeeping and DNA repair genes, including *ligD* and *ku*. The surrounding loci, including multiple genes encoding glycopeptide synthesis or secretion-related proteins (*gspG*–*gspL*–*gspD*–*gspE*), were structurally preserved between the two genomes, showing extensive nucleotide identity across the aligned segment (green shading). Only minor variations in intergenic spacing or small hypothetical ORFs were observed.

**Figure 2:**
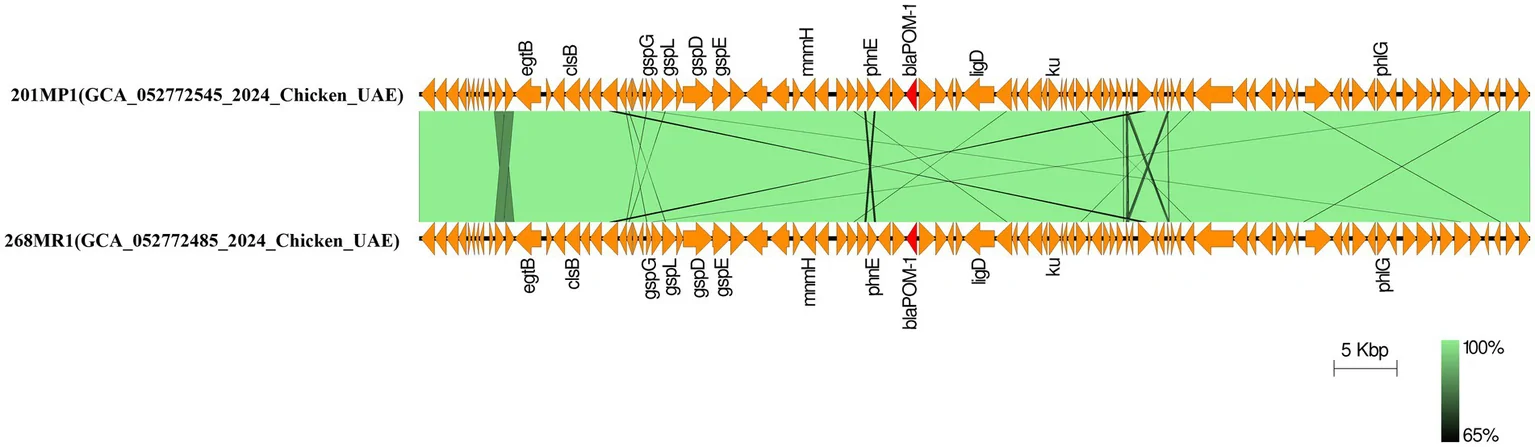
Comparative genetic environment of the chromosomal *bla*_POM-1_ gene in *Metapseudomonas otitidis* isolates 201MP1 and 268MR1. The regions flanking *bla*_POM-1_ show high synteny and nucleotide identity between both isolates (green shading). In both genomes, *bla*_POM-1_ is located downstream of the conserved *mmhH*–*phnE* region and flanked by core chromosomal genes (*ligD* and *ku*), supporting its intrinsic and conserved chromosomal nature in *M. otitidis*.

### 3.5 Phylogenomic analysis

Phylogenomic analysis of 538 publicly available *K. pneumoniae* ST11 isolates carrying the *bla*_NDM-5_ gene revealed that isolate 377MR1 clustered tightly within a well-supported clade of globally distributed CRKP strains. Among the 538 *K. pneumoniae* ST11 isolates carrying *bla*_NDM-5_, more than half originated from Asia (316 isolates, 58.7%), followed by Europe (162 isolates, 30.1%), with smaller contributions from South America, North America, Africa, and Oceania (Figure 3A). China was the primary source, accounting for 271 isolates (50.4%), while other countries such as Poland, Greece, Brazil, and Pakistan contributed far fewer isolates. These findings show that *bla*_NDM-5_-positive ST11 strains are strongly concentrated in Asia, particularly China, with additional cases reported across multiple continents.

**Figure 3:**
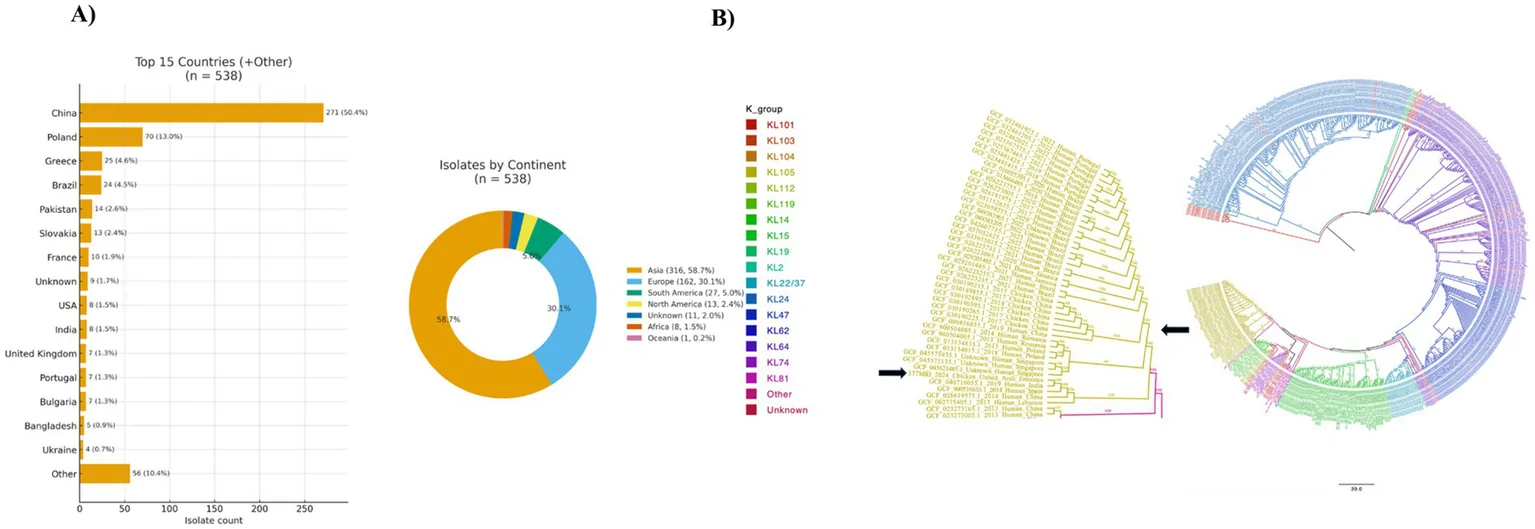
Global distribution and phylogenomic relationships of *bla*_NDM-5_-carrying *Klebsiella pneumoniae* ST11 isolates (*n* = 538). **(A)** Geographic distribution of *K. pneumoniae* ST11 isolates carrying *bla*_NDM-5_ obtained from GenBank (*n* = 538) with more than half the strains originating from Asia. **(B)** Maximum-likelihood phylogenomic tree depicting the evolutionary relationships among the 538 ST11 isolates. Branches are colored according to capsular polysaccharide loci (KL groups), highlighting the substantial capsular diversity within the ST11 lineage. Major KL types, including KL24, KL64, KL47, KL15, and KL105, are distributed across multiple phylogenetic clades. The study isolate (377MR1) clusters within a well-supported KL105 clade, together with closely related isolates from Asia, illustrating the global dissemination of this successful capsular lineage. Scale bar indicates nucleotide substitutions per site.

Analysis of capsular polysaccharide loci (KL types) revealed substantial capsular diversity within the ST11 population, underscoring the evolutionary flexibility of this high-risk clone. Although multiple KL groups were detected—including KL24, KL64, KL47, KL15, and KL105—KL105 emerged as one of the dominant capsular types among *bla*_NDM-5_-positive ST11 isolates (Figure 3B). Notably, isolate 377MR1 belonged to the KL105 group, placing it within a prevalent capsular lineage that has been reported across multiple geographic regions. The coexistence of diverse KL types within ST11 suggests frequent capsular switching events, which may facilitate immune evasion, environmental adaptation, and sustained global spread of carbapenem-resistant ST11 lineages.

Comparative whole-genome SNP-based reconstruction demonstrated that 377MR1 shared the highest genomic similarity with an isolate recovered from a human patient in India in 2019 (GCF_040716055.1), indicating a close evolutionary relationship and suggesting a possible shared lineage or dissemination pathway (Figure 3B). The ST11 *bla*_NDM-5_ genomes formed several geographic sub-clusters, yet 377MR1 fell within a predominantly Asian clade that included clinical isolates from India, China, and Lebanon.

Phylogenomic analysis of *M. otitidis* isolates 201MP1 and 268MR1, together with other 42 *M. otitidis* draft genomes recovered from different sources worldwide, demonstrated that both isolates clustered closely together (Figure 4). Whole-genome SNP-based comparison placed the two UAE chicken isolates within the same well-supported lineage, confirming their strong genetic relatedness. Notably, this clade also included a *M. otitidis* genome recovered from a human host in the United States in 2016 (GCF_900111283.1), suggesting that these isolates may belong to a globally distributed sub-lineage with the potential for cross-host and cross-regional dissemination.

**Figure 4:**
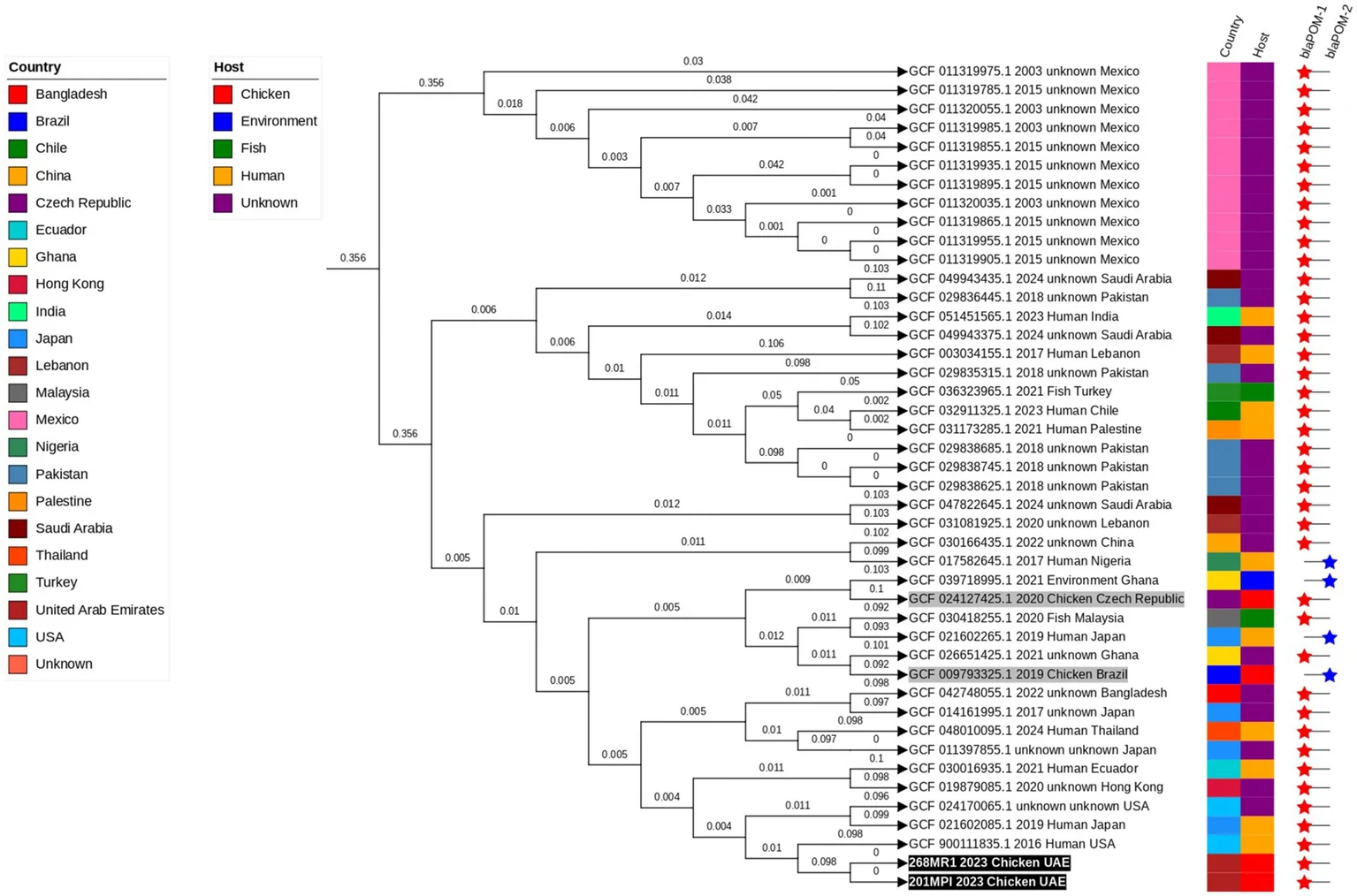
Phylogenomic relationships of *bla*_POM-1_–producing *Metapseudomonas otitidis*. The tree is based on whole-genome SNP analysis of 44 *M. otitidis* genomes, including UAE chicken isolates 201MP1 and 268MR1 and 42 publicly available genomes. Colored bars indicate the country of origin and host source. Both UAE isolates cluster closely within the same lineage, which also includes a human isolate from the USA (2016), suggesting a globally distributed sub-lineage.

## 4 Discussion

Carbapenemase-producing bacteria represent a major global AMR threat, with the WHO classifying carbapenem-resistant Enterobacterales as critical-priority pathogens due to their rapid spread and limited treatment options (3–8). Although traditionally linked to human healthcare settings, accumulating evidence shows that food-producing animals and agricultural environments can also act as reservoirs for clinically important carbapenemase genes, including *bla*_NDM,_ *bla*_VIM,_ and *bla*_OXA-48-like_ (3, 5–7). Against this backdrop, our study provides important One Health insights by examining carbapenemase-producing bacteria within poultry production systems in the UAE, a setting where such data remain limited.

In the present study, screening on meropenem-supplemented MacConkey agar, followed by phenotypic confirmation using the modified carbapenem inactivation method (mCIM), identified three isolates (3.9%) as carbapenemase producers. This finding underscores the importance of confirmatory testing, as reduced susceptibility to carbapenems may arise from mechanisms other than carbapenemase production, including porin loss and efflux pump overexpression (14). These three confirmed carbapenemase-producing isolates exhibited resistance to multiple antimicrobial classes, with two isolates (268MR1 and 377MR1) meeting the criteria for multidrug resistance. Whole-genome sequencing revealed important distinctions among these isolates. Only one isolate, *K. pneumoniae* 377MR1, carried a clinically significant acquired carbapenemase gene (*bla*_NDM-5_), whereas the remaining two isolates were identified as *M. otitidis* harboring the chromosomal carbapenemase *bla*_POM-1_, which is intrinsic to the species. It is worth mentioning that *bla*_NDM_-producing *K. pneumoniae* and other Enterobacterales have been identified in humans in the UAE, highlighting the regional circulation of this clinically important resistance determinant. Previous reports from clinical settings in the UAE have documented the presence of *bla*_NDM-5_ in *K. pneumoniae* often in association with diverse plasmid replicons and additional β-lactamase genes, underscoring the complexity and clinical significance of these resistance profiles (14). The detection of *bla*_NDM-5_ in a poultry-associated *K. pneumoniae* isolate is particularly concerning, as *bla*_NDM-5_ has been reported in human clinical isolates in the UAE and the wider region, frequently in combination with other β-lactamases such as SHV, as demonstrated in earlier PCR-based studies prior to the routine use of whole-genome sequencing (15).

Notably, *bla*_NDM-5_ has also been reported in *E. coli* from human infections in the UAE, supporting its dissemination across Enterobacterales and reinforcing the relevance of a One Health perspective when interpreting its emergence in animal reservoirs (16). Of additional concern, *bla*_NDM-5_ has been detected in strains resistant to newly introduced, last-resort antimicrobial agents, further highlighting the public health risk associated with this gene and the potential consequences of its establishment in food-producing animals with opportunities for zoonotic or environmental transmission (17). Taken together, the identification of *bla*_NDM-5_in a poultry-derived *K. pneumoniae* isolate, alongside accumulating evidence from human clinical studies in the UAE, raises the possibility of human-to-animal spillover, shared environmental reservoirs or other indirect transmission pathways, facilitating the dissemination of healthcare-associated resistance determinants. These findings reinforce the critical need for continuous phenotypic and genomic surveillance across human, animal, and environmental interfaces to better understand the transmission dynamics of carbapenemase-producing Enterobacterales and their contribution to the broader antimicrobial resistance ecosystem.

The genomic characterization of the carbapenemase-producing isolates revealed marked diversity in their resistomes, reflecting the complex array of resistance mechanisms that may circulate in poultry production environments. The identification of *bla*_NDM-5_ in the *K. pneumoniae* ST11 isolate is particularly significant, as NDM-5 is strongly associated with globally disseminated high-risk clones in human clinical settings and has been increasingly reported as a key driver of carbapenem resistance (18, 19). Its co-occurrence with multiple β-lactamase genes and a broad panel of resistance determinants against aminoglycosides, quinolones, tetracyclines, sulfonamides, macrolides, fosfomycin, and phenicols underscores the extensively multidrug-resistant nature of this strain, consistent with previous reports of ST11 as a major international MDR lineage (18, 19). Furthermore, the identification of two *M. otitidis* isolates carrying the intrinsic carbapenemase *bla*_POM-1_ and *bla*_LCR-1_, in addition to the presence of mobile resistance genes—including *tmexD4*, *qnrVC1*, *sul1*, and *aadA10*—highlights the capacity of environmental non-fermenters to accumulate clinically relevant resistance determinants. The emergence of *tmexD* family efflux pump genes is particularly concerning given their role in reducing susceptibility to tigecycline and multiple other drug classes (20).

The combined plasmid, virulence, and genomic analyses provide a comprehensive view of the potential public health risks associated with carbapenemase-producing bacteria circulating in poultry production environments. The detection of multiple plasmid replicons—IncFIB(K), IncFII(K), IncR, and Col(MG828)—in *K. pneumoniae* 377MR1 is notable, as these plasmid families are frequently associated with high-risk clones and the mobilization of antimicrobial resistance genes, particularly *bla*_NDM_ variants (21). Their absence in *M. otitidis* isolates suggests that resistance in these strains is predominantly chromosomal rather than plasmid-mediated. The virulence gene repertoire of *K. pneumoniae* 377MR1, including a complete yersiniabactin locus, enterobactin system, *ompA*, and an ECP-like pilus cluster, reflects a pathogenic profile consistent with successful clinical lineages and is known to enhance iron acquisition, epithelial adherence, serum resistance, and biofilm formation (22, 23). In contrast, both *M. otitidis* isolates possessed conserved virulence architectures dominated by a full Type VI secretion system, pyoverdine siderophore cluster, flagellar motility genes, and alginate biosynthesis machinery—features characteristic of environmental *Pseudomonas* species associated with persistence, host colonization, and environmental fitness (24).

Genomic context analysis further highlighted the mobility potential of *bla*_NDM-5_, which was embedded in a highly conserved IS*30*–*bla*_NDM_–*ble*_MBL_ resistance cassette in isolate 377MR1, closely matching a human *K. pneumoniae* isolate from India recovered in 2019. The high degree of synteny and >98% nucleotide identity underscores the stability and global dissemination of this mobile element across geographically distinct ST11 lineages (25). Furthermore, the high degree of structural conservation observed in the *bla*_NDM-5_ genetic environment reflects the widespread dissemination of a stable mobile resistance platform rather than direct epidemiological linkage between isolates. Conversely, the *bla*_POM-1_ carbapenemase in *M. otitidis* was located within a stable chromosomal region shared between the two isolates, consistent with reports describing *bla*_POM_ as an intrinsic, vertically inherited carbapenemase in this species (24, 26). Phylogenomic comparisons further reinforced these findings, as the NDM-5–producing *Klebsiella pneumoniae* isolate clustered tightly with ST11 genomes originating predominantly from Asia—particularly India and China—highlighting the ongoing global expansion of this epidemic lineage (27). Importantly, this phylogenetic relatedness was mirrored at the capsular level: both our isolate and closely related Indian strains belonged to the KL105 capsular group, one of the dominant KL types among *bla*_NDM-5_-positive ST11 isolates. This observation underscores the successful international dissemination of a specific ST11 KL105 sublineage, rather than independent local emergence events.

Previous studies have demonstrated that the ST11 KL105 clade has disseminated widely in Europe, predominantly as ESBL- or DHA-1–producing isolates, while maintaining extensive variability in plasmid backgrounds and β-lactamase content (28). Our findings expand this epidemiological framework by showing that the same ST11 KL105 lineage can also acquire *bla*_NDM-5_, further supporting the notion that this capsular background provides a stable genomic scaffold for the accumulation of diverse resistance determinants. Additionally, the first reports of ST11 KL105 isolates harboring *bla*_KPC-3_ in Latin America reinforce the global success and adaptability of this lineage (29).

Taken together, the observed capsular diversity within ST11, coupled with the repeated emergence of KL105-associated high-risk subclades, suggests that both capsular switching and the success of particular capsular lineages contribute to the persistence and worldwide spread of carbapenem-resistant ST11 *K. pneumoniae*. The recurrent association of KL105 with distinct carbapenemases—including KPC, NDM-1, and NDM-5—highlights this capsular type as a key epidemiological marker and underscores the value of incorporating capsular typing into genomic surveillance and infection control strategies. Furthermore, the *M. otitidis* isolates formed a highly cohesive clade and were closely related to a human isolate from the United States recovered in 2016, suggesting the emergence of a potentially widespread sub-lineage with possible cross-host or zoonotic relevance. Although standard microbiological and genomic approaches were applied, this study extends our previous work by providing the first integrated phenotypic and phylogenomic characterization of carbapenemase-producing bacteria from poultry farms in the United Arab Emirates, revealing genomic links to human clinical isolates, while emphasizing that these findings represent exploratory surveillance and highlighting the need for future studies with systematic isolate-level traceability, environmental controls, and longitudinal sampling to confirm true on-farm colonization versus potential post-harvest contamination.

This study has some limitations. First, the relatively small sample size and low number of carbapenemase-producing isolates reflect the exploratory nature of this investigation and preclude robust estimates of the prevalence of carbapenemase-producing bacteria in the UAE poultry industry. Second, sampling was restricted to broiler farms belonging to two commercial poultry companies in Al Ain, limiting the epidemiological representativeness of the findings and their generalizability to poultry production systems across the country. Third, although standard aseptic procedures were implemented throughout sampling and laboratory processing, formal contamination controls (e.g., field blanks and sequencing audits) were not systematically included, and therefore low-level contamination cannot be completely excluded. The use of short-read whole-genome sequencing limited complete resolution of plasmids and complex resistance regions, preventing definitive determination of the genetic location and mobility of some resistance determinants; future studies incorporating long-read sequencing with complementary experimental approaches, including plasmid profiling, S1-PFGE, and conjugation assays, will provide improved genomic resolution. In addition, although antimicrobial susceptibility testing followed CLSI quality control recommendations using *E. coli* ATCC 25922, future studies will incorporate additional quality control strains, including those recommended for non-fermenting Gram-negative bacteria, to further strengthen phenotypic quality assurance. Finally, although this study was conducted within a One Health framework, concurrent sampling of farm workers, poultry, and environmental reservoirs was not performed. Consequently, while comparative genomic analyses demonstrate close evolutionary relatedness between the poultry isolates and previously reported human clinical strains, they do not permit inference of direct cross-host transmission, transmission pathways, or plasmid dissemination. Future large-scale, longitudinal One Health studies integrating human, animal, food, and environmental sampling are therefore warranted to better elucidate the epidemiology and transmission dynamics of carbapenemase-producing bacteria.

## 5 Conclusion

This study provides important early warning signal rather than a prevalence estimate from One Health insights into the occurrence and genomic characteristics of carbapenemase-producing bacteria within poultry production systems in the UAE. Furthermore, this work represents to our knowledge the first integrated phenotypic and genomic characterization of carbapenemase-producing bacteria from poultry production systems in the United Arab Emirates. Beyond reporting their occurrence, the study characterizes resistance and virulence determinants, investigates the genetic context of carbapenemase genes, and places the isolates within a global phylogenomic framework. These comprehensive analyses provide novel insights into the evolution and dissemination of clinically important resistance determinants and therefore exceed the scope of a conventional short communication. Although only three isolates were confirmed as true carbapenemase producers, whole-genome sequencing revealed the presence of a high-risk *K. pneumoniae* ST11 strain carrying *bla*_NDM-5_ alongside multiple plasmid-borne antimicrobial resistance and virulence determinants, consistent with the global dissemination of this lineage. The conserved genetic environment of *bla*_NDM-5_ and its close phylogenomic relatedness to a human clinical isolate from India highlight the potential for regional or international dissemination of this globally significant lineage. In contrast, *M. otitidis* isolates harbored the intrinsic carbapenemase *bla*_POM-1_ and displayed highly conserved chromosomal virulence and resistance features, clustering closely with a previously reported human isolate. Together, these findings underscore the capacity of poultry-associated bacteria to carry clinically relevant resistance genes and virulence factors, reinforcing the need for continuous genomic surveillance to better understand transmission dynamics and to mitigate the spread of high-risk antimicrobial resistance determinants across human, animal, and environmental sectors.

## Data Availability

The datasets presented in this study can be found in online repositories. The names of the repository/repositories and accession number(s) can be found: https://www.ncbi.nlm.nih.gov/genbank/, SAMN51336938, SAMN51336939, and SAMN51336940.

## Footnotes

1
https://github.com/jekesa

2
https://github.com/tseemann/abricate

3
https://usegalaxy.org.au

4
https://github.com/tseemann/snippy

5
https://www.evolgenius.info/evolview/
